# scPipe: A flexible R/Bioconductor preprocessing pipeline for single-cell RNA-sequencing data

**DOI:** 10.1371/journal.pcbi.1006361

**Published:** 2018-08-10

**Authors:** Luyi Tian, Shian Su, Xueyi Dong, Daniela Amann-Zalcenstein, Christine Biben, Azadeh Seidi, Douglas J. Hilton, Shalin H. Naik, Matthew E. Ritchie

**Affiliations:** 1 Molecular Medicine Division, The Walter and Eliza Hall Institute of Medical Research, Parkville, Australia; 2 Department of Medical Biology, The University of Melbourne, Parkville, Australia; 3 College of Life Science, Zhejiang University, Hangzhou, Zhejiang Province, P.R. China; 4 Australian Genome Research Facility, Parkville, Australia; 5 School of Mathematics and Statistics, The University of Melbourne, Parkville, Australia; Johns Hopkins University, UNITED STATES

## Abstract

Single-cell RNA sequencing (scRNA-seq) technology allows researchers to profile the transcriptomes of thousands of cells simultaneously. Protocols that incorporate both designed and random barcodes have greatly increased the throughput of scRNA-seq, but give rise to a more complex data structure. There is a need for new tools that can handle the various barcoding strategies used by different protocols and exploit this information for quality assessment at the sample-level and provide effective visualization of these results in preparation for higher-level analyses. To this end, we developed *scPipe*, an R/Bioconductor package that integrates barcode demultiplexing, read alignment, UMI-aware gene-level quantification and quality control of raw sequencing data generated by multiple protocols that include CEL-seq, MARS-seq, Chromium 10X, Drop-seq and Smart-seq. *scPipe* produces a count matrix that is essential for downstream analysis along with an HTML report that summarises data quality. These results can be used as input for downstream analyses including normalization, visualization and statistical testing. *scPipe* performs this processing in a few simple R commands, promoting reproducible analysis of single-cell data that is compatible with the emerging suite of open-source scRNA-seq analysis tools available in R/Bioconductor and beyond. The *scPipe* R package is available for download from https://www.bioconductor.org/packages/scPipe.

## Author summary

Biotechnologies that allow researchers to measure gene activity in individual cells are growing in popularity. This has resulted in an avalanche of custom analysis methods designed to deal with the complex data that arises from this technology. Although hundreds of analysis methods are available, relatively few deal with raw data processing in a holistic way. Our *scPipe* software has been developed to fill this gap. *scPipe* is the first fully integrated R package that deals with the raw sequencing reads from single cell gene expression studies, processing them to the point where biologically interesting downstream analyses can take place. By following community developed standards, *scPipe* is compatible with many other software packages for single cell analysis available from the open-source Bioconductor project, facilitating a complete beginning to end analysis of single cell gene expression data. This allows various biological questions to be answered, ranging from the identification of novel cell types to the discovery of new marker genes. *scPipe* promotes reproducibility and makes it easier for researchers to share results and code.

This is a *PLOS Computational Biology* Software paper.

## Introduction

Advances in single-cell transcriptomic profiling technologies allow researchers to measure gene activity in thousands of cells simultaneously, enabling exploration of gene expression variability [1], identification of new cell types [2] and the study of transcriptional programs involved in cell differentiation [3]. The introduction of cellular barcodes, sequences distinct for each cell attached to the dT-primer, has increased the throughput and substantially reduced the cost of single-cell RNA sequencing (scRNA-seq). These barcodes allow for the demultiplexing of reads after cells are pooled together for sequencing. Apart from cellular barcodes, molecular barcodes or unique molecular identifiers (UMIs), are frequently employed to remove PCR duplicates and allow identification of unique mRNA molecules, thereby reducing technical noise. The multiple levels of barcoding used in scRNA-seq experiments create additional challenges in data processing together with new opportunities for quality control (QC). Different protocols use different barcode configurations, which means a flexible approach to data preprocessing is required.

A large number of software tools have already been tailored to scRNA-seq analysis [4], the majority of which are focused on downstream tasks such as clustering and trajectory analysis. Methods for preprocessing tend to focus on specific tasks such as *UMI-tools* [5], *umitools* (http://brwnj.github.io/umitools/) and *umis* [6] which have been developed for handling random UMIs and correcting UMI sequencing errors. Other tools such as *CellRanger* [7], *dropEst* [8] and *dropseqPipe* (https://github.com/Hoohm/dropSeqPipe) on the other hand offer a complete preprocessing solution for data generated by droplet based protocols. Other packages such as *scater* [9], and *scran* [10] work further downstream by preprocessing the counts to perform general QC and normalization of scRNA-seq data.

*scPipe* was developed to address the lack of a comprehensive R-based workflow for processing sequencing data from different protocols that can accommodate both UMIs and sample barcodes, map reads to the genome and summarise these results into gene-level counts. Additionally this pipeline collates useful metrics for QC during preprocessing that can be later used to filter genes and samples. In the remainder of this article we provide an overview of the main features of our *scPipe* software and demonstrate its use on various in-house generated and publicly available scRNA-seq datasets.

## Design and implementation

### Single-cell RNA-seq datasets analysed

#### Mouse hematopoietic lineage dataset

Single cell expression profiling of the main hematopoietic lineages in mouse (erythroid, myeloid, lymphoid, stem/progenitors) was performed using a modified CEL-seq2 [11] protocol. B lymphocytes (B220+ FSC-Alow), erythroblasts (Ter119+ CD44+, FSC-Amid/high), granulocytes (Mac1+ Gr1+) and high-end progenitor/stem (Lin- Kit+ Sca1+) were sorted from the bone marrow of a C57BL/6 10-13 week old female mouse. T cells (CD3+ FSC-Alow) were isolated from the thymus of the same mouse. Bone marrow and thymus were dissociated mechanically, washed and stained with antibodies for 1hr on ice. Single cells were deposited on a 384 well plate using an Aria cell sorter (Beckman). Index data was collected and our adapted CEL-seq2 protocol used to generate a library for sequencing. The reads were sequenced by an Illumina Nextseq 500 and processed by *scPipe*. This dataset is available under GEO accession number GSE109999.

#### Human lung adenocarcinoma cell line dataset

The cell lines H2228, NCI-H1975 and HCC827 were retrieved from ATCC (https://www.atcc.org/) and cultured in Roswell Park Memorial Institute (RPMI) 1640 medium with 10% fetal calf serum (FCS) and 1% Penicillin-Streptomycin. The cells were grown independently at 37°C with 5% carbon dioxide until near 100% confluency. Cells were PI stained and 120,000 live cells were sorted for each cell line by FACS to acquire an accurate equal mixture of live cells from the three cell lines. This mixture was then processed by the Chromium 10X single cell platform using the manufacturer’s (10X Genomics) protocol and sequenced with an Illumina Nextseq 500. Filtered gene expression matrices were generated using *CellRanger* (10X Genomics) and *scPipe* independently. For *CellRanger*, we used the default parameters, with --expect-cells = 4000. For *scPipe*, we processed the 4,000 most enriched cell barcodes. The GRCh38 human genome and ENSEMBL v91 human gene annotation were used for both the *scPipe* and *CellRanger* analysis. In *scPipe*, the number of components used in the outlier detection step was set to comp = 2 for quality control to remove poor quality cells. This dataset is available under GEO accession number GSE111108.

#### Ischaemic sensitivity of human tissue dataset

A publicly available Chromium 10X dataset from the Human Cell Atlas project (https://preview.data.humancellatlas.org/) was also preprocessed using *scPipe* and analysed with highly compatible Bioconductor packages. This dataset of roughly 2,000 cells comes from the first spleen harvested in a project seeking to study sensitivity to ischaemia in 3 different tissue types [12]. For this sample, *scPipe* was run with quality and sequence filters turned off in sc_trim_barcode and cellular barcodes were detected from a sample of the first 5 million reads using sc_detect_bc, which resulted in 2,273 detected barcodes. The sequences were aligned to the GRCh38 human genome using *Rsubread* and counts were assigned based on ENSEMBL v91 human gene annotations using *scPipe* and outliers were detected with comp = 2 in the automatic outlier detection step.

### *scPipe* implementation details

*scPipe* is an R [13] / Bioconductor [14] package that can handle data generated from all popular 3’ end scRNA-seq protocols and their variants, such as CEL-seq, MARS-seq, Chromium 10X and Drop-seq. Data from non-UMI protocols generated by Smart-seq and Smart-seq2 can also be handled. The pipeline begins with FASTQ files and outputs both a gene count matrix and a variety of QC statistics. These are presented in a standalone HTML report generated by *rmarkdown* [15] that includes various plots of QC metrics and other data summaries. The *scPipe* package is written in R and C++ and uses the *Rcpp* package [16, 17] to wrap the C++ code into R functions and the *Rhtslib* package [18] for BAM input/output. The key aspects are implemented in C++ for efficiency.

## Results

### The *scPipe* workflow

#### FASTQ reformatting

**Fig 1:**
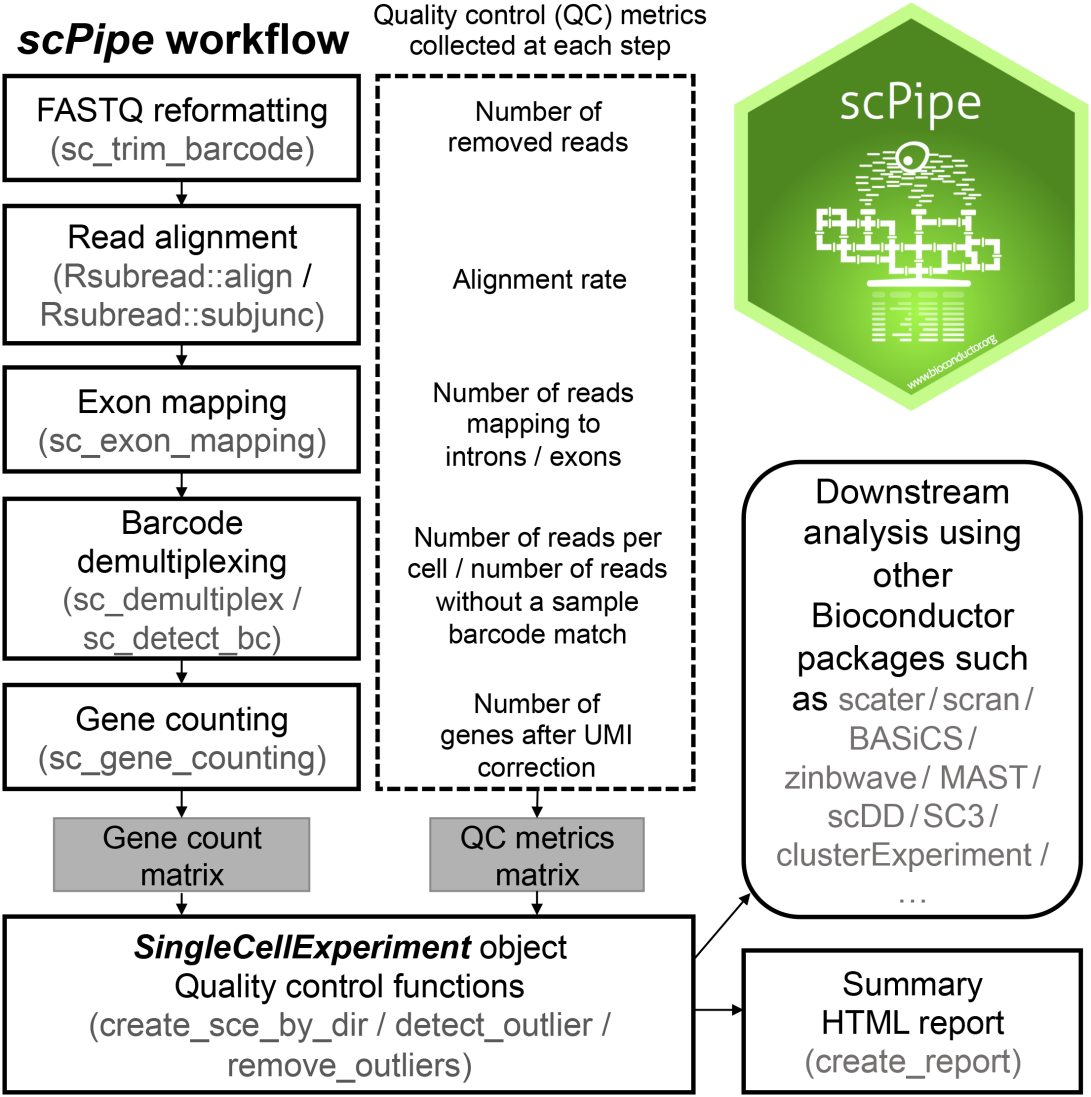
The *scPipe* workflow. *scPipe* is an R/Bioconductor package that uses functionality from a number of other packages, including *Rsubread* to align reads to a reference genome (although in practice any aligner that produces BAM files can be used for read alignment) and *SingleCellExperiment* to organise the counts and sample annotation information. The major steps in the preprocessing pipeline of *scPipe* are shown along with the quality control (QC) statistics collected at each stage. The final output of this process is a matrix of counts and QC metrics for use in downstream analysis and an HTML report that summarises the analysis. The resulting *SingleCellExperiment* object can be used as input to other Bioconductor packages to perform further downstream analysis. *scPipe* logo created by Roberto Bonelli published under a CC0 1.0 license (https://github.com/Bioconductor/BiocStickers/blob/master/scPipe/README.md).

The *scPipe* workflow (Fig 1) generally begins with paired-end FASTQ data which is passed to the function sc_trim_barcode, which reformats the reads by trimming the barcode and UMI from the reads and moving this information into the read header. There are options to perform some basic filtering in this step, including removing reads with low quality sequence in the barcode and UMI regions and filtering out low complexity reads, most of which are non-informative repetitive sequences such as polyA. The output FASTQ file contains transcript sequences, with barcode information merged into the read names.

#### Read alignment, exon mapping and barcode demultiplexing

The next stage of preprocessing involves sequence alignment. For this task, any popular RNA-seq read aligner that produces BAM formatted output can be used. By default, *scPipe* uses the fast and convenient R-based *Rsubread* [19] aligner which is available on Linux and Mac OS operating systems. For publicly available datasets from sources such as GEO, FASTQ files are often available per cell and these need to be aligned first before they can be processed further using *scPipe*. For all different types of data, aligned reads in the BAM file are then assigned to exons by the sc_exon_mapping function according to a user provided annotation. Using a similar strategy to *featureCounts* [20], we divide chromosomes into non-overlapping bins and assess the overlap of aligned fragments with exons that fall within the bin to reduce the search complexity. This function records the mapping result, together with the UMI and cell barcodes available from the optional fields of the BAM file with specific BAM tags. By default we use the official BAM tag BC for cell barcode and OX for UMI sequence.

**Fig 2:**
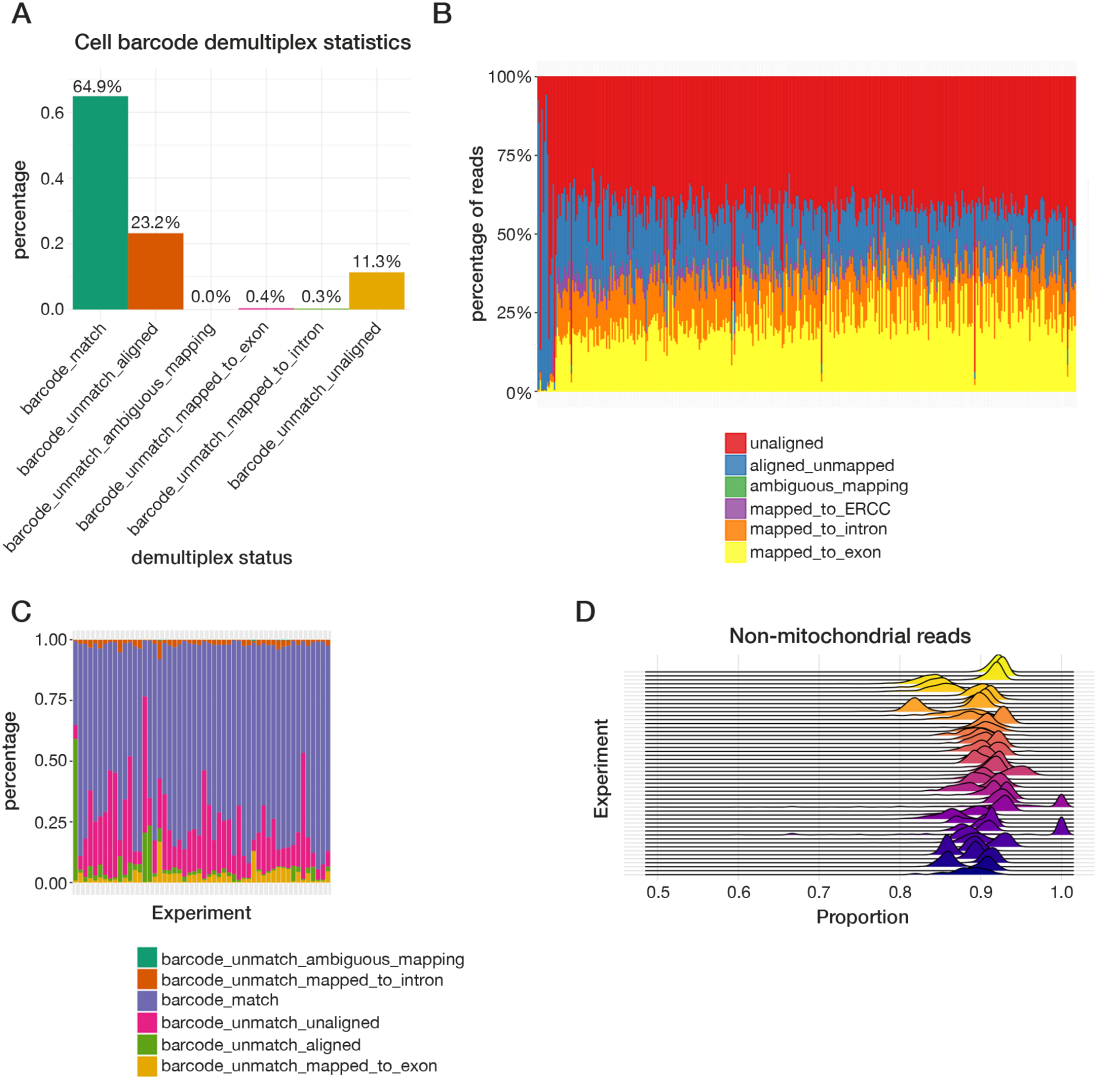
Example QC plots that can be created using output from *scPipe* to assess data quality both within and between experiments. Within experiment displays include (**A**) a bar plot illustrating the overall cell barcode matching results to assess sequencing accuracy across all samples and (**B**) a stacked bar plot showing the mapping rate, separated into reads that map to exon, intron and ERCCs and those that are ambiguously mapped, map elsewhere in the genome and are unaligned for each cell in an experiment (ordered by exon mapping rate). Between experiment displays include (**C**) a stacked bar plot showing the cell barcode matching results from panel (A) from multiple experiments and (**D**) a ridgeline plot presenting the distribution of proportions of non-mitochondrial read counts for cells across multiple experiments.

Next, the sc_demultiplex function is used to demultiplex results per cell using the sample barcode information. For CEL-seq and MARS-seq, the demultiplexing is based on the list of designed barcode sequences that the user provides, and allows for mismatches during the cell barcode matching step. For 10X and Drop-seq where the cell barcode sequences need to be identified from the reads, the function sc_detect_bc can be applied to identify enriched barcodes in the data that can then be supplied to sc_demultiplex. This function can accept a list of possible barcodes that may be present in the dataset (if available), such as the white list provided by 10X. Data from non-UMI protocols such as Smart-seq and Smart-seq2 can be handled by setting has_UMI to FALSE when running sc_demultiplex. The sc_detect_bc function summarises the sequences in the cell barcode region, collapsing reads with a small edit distance, which are indicative of sequencing errors. It can report a given number of cell barcodes or keep the cell barcodes based on a read number cutoff. A relaxed threshold is generally recommended when one specifies the number of barcodes to retain, for instance if the estimated number of cells in the library is 1,000-5,000, a threshold of 10,000 would ensure data for all cells is collected. The motivation behind this is that unlike *CellRanger*, we don’t recommend detecting low quality cells or empty barcodes solely based on the number of reads or UMI counts per cell. Instead, more robust and meaningful results can be produced using multiple QC metrics. Hence the sc_detect_bc function should be run to allow more barcodes than cells. The quality control function in *scPipe* can be used to remove erroneous cell barcodes at a later stage. The demultiplexed data are output in a csv file for each cell, where each row corresponds to a read that maps to a specific gene, and columns that include gene id, UMI sequence and mapping position. The overall barcode demultiplexing results are recorded and can be plotted as shown in Fig 2A for the mouse blood cells generated by the CEL-seq2 protocol.

#### Obtaining a gene count matrix and performing quality assessment

The next stage of preprocessing makes use of the sc_gene_counting function to remove PCR duplicates (also known as UMI deduplication) to generate a gene count matrix. *scPipe* uses a greedy approach based on hamming distance to do this. The detected UMIs are compared to each other and if two UMI sequences are found to be within a certain hamming distance apart (the default is 1) and one UMI has more than twice the count of the other, then the two different UMIs will be treated as instances of the same UMI. The approach is based on the fact that erroneous UMI sequences due to sequencing error usually just have one or two reads. PCR errors, however, can be more problematic, and other more sophisticated network-based methods such as the approach taken in *UMI-tools* have been developed to deal with such errors. When PCR error rates are low, the approach used in *scPipe* should give good results. Our pipeline outputs the raw UMI sequences that map to each gene, which can be used as input for more sophisticated UMI correction methods when the PCR error rate is high. After UMI deduplication we get a gene count matrix that can be used for further analysis. QC information is collected at each step (Fig 1) and includes the total number of mapped reads per cell, UMI deduplication statistics, per cell barcode demultiplexing statistics, UMI correction statistics and External RNA Controls Consortium (ERCC) spike-in control statistics (where present).

*scPipe* uses the S4 infrastructure provided by the *SingleCellExperiment* package [21] for data storage. A SingleCellExperiment object can be constructed by the create_sce_by_dir function using the data folder generated during preprocessing, or by manually specifying all the required slots. Alongside the gene count matrix, this object stores QC information obtained during preprocessing, and the type of gene id and organism name, which can both be useful in downstream functional enrichment analysis.

**Fig 3:**
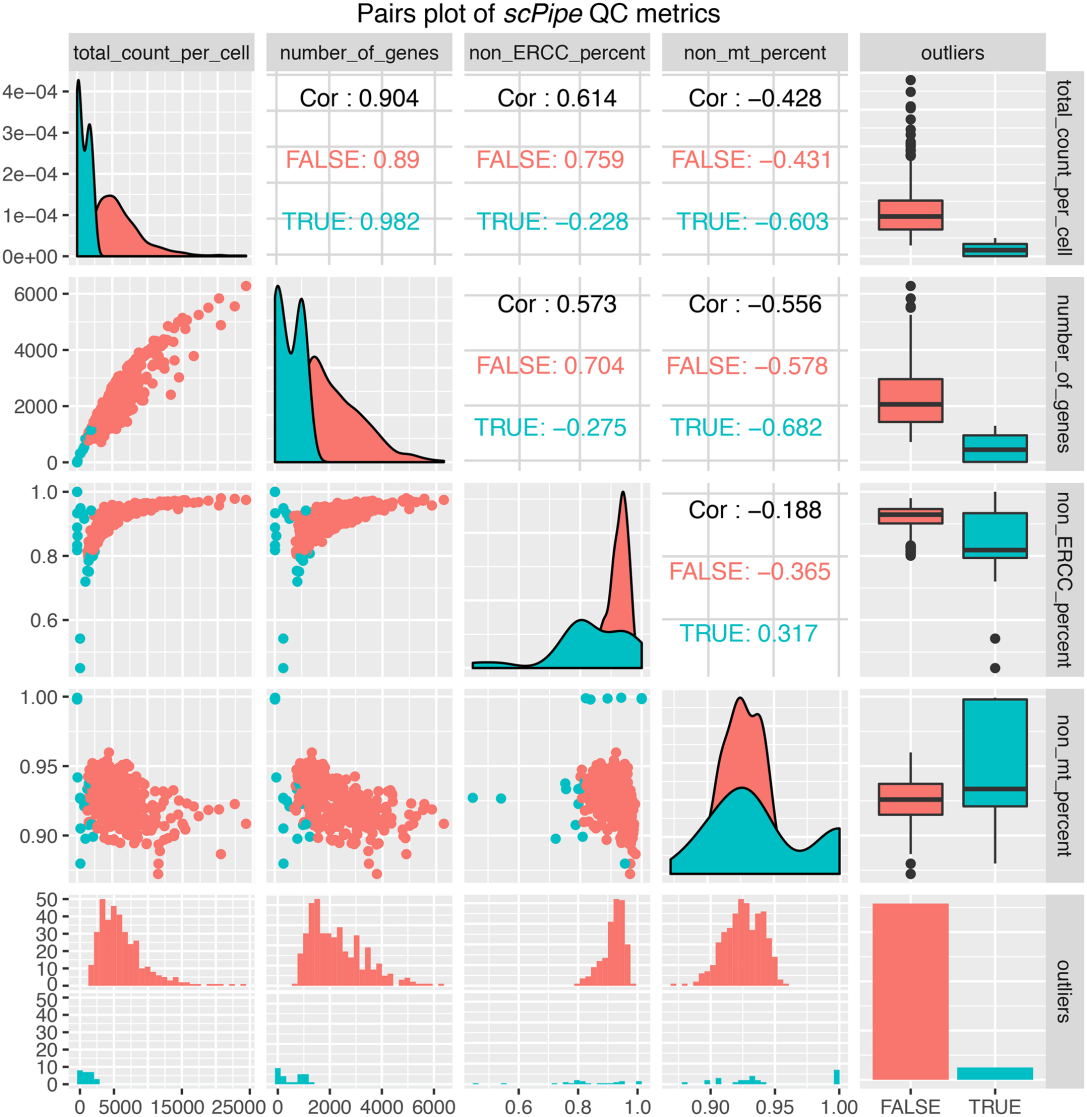
A pairwise scatter plot of the quality control metrics collected by *scPipe* for the mouse blood cell dataset. Sample-specific metrics include the total read count, the number of genes detected and the proportion of non-ERCC or non-mitocondrial reads. The good quality and outlier samples detected by *scPipe*’s automatic outlier detection method are indicated in each panel by a different colour.

Next, alignment statistics for each cell can be plotted using the plotMapping function (Fig 2B). Data shown here is again from the mouse blood cell dataset described previously. Monitoring these QC statistics across experiments (Fig 2C and 2D) can be particularly useful for labs that routinely process single-cell data, allowing them to assess the impact changes in lab processes and protocols have on data quality. Pairwise scatter plots of QC metrics such as the total molecules per cell and the number of genes detected can be generated with the plotQC_pair function (Fig 3).

*scPipe* implements a multivariate outlier detection method for discovering low quality cells to remove from further analysis. The method uses up to 5 metrics (log-transformed number of molecules detected, the number of genes detected, the percentage of reads mapping to ribosomal, or mitochondrial genes and ERCC recovery (where available)) as input (Fig 3) to a Gaussian mixture model (GMM). There are three stages in the outlier detection process. First the Mahalanobis distances between cells based on the quality control metrics chosen are calculated, with extreme cells removed before fitting the GMM using the *mclust* package [22]. The QC metrics of the remaining cells are used to capture the overall heterogeneity in the dataset. The mixture component with systematically lower QC metrics, such as lower numbers of genes detected and lower numbers of molecular counts are marked as outliers. In the final stage, the Mahalanobis distance is calculated for cells from the mixture component with the highest QC metric values with outliers automatically detected based on this distance. Outlier detection can then be reconciled with visual inspection of the QC metrics through the afore-mentioned QC plotting options to fine tune the sample-specific filtering thresholds chosen. Depending on the data, the only argument that needs to be specified is the maximum number of components in the GMM. In most cases, this is set to 1 or 2 for good quality data with only a small proportion of poor quality cells. For data generated by droplet-based protocols where larger proportions of poor quality cells may be expected, the number of components can be set to 2 or 3 to model extra components that correspond to the good and poor quality cells. Apart from the number of components, the user can also adjust the confidence interval for choosing outliers (0.99 by default).

**Fig 4:**
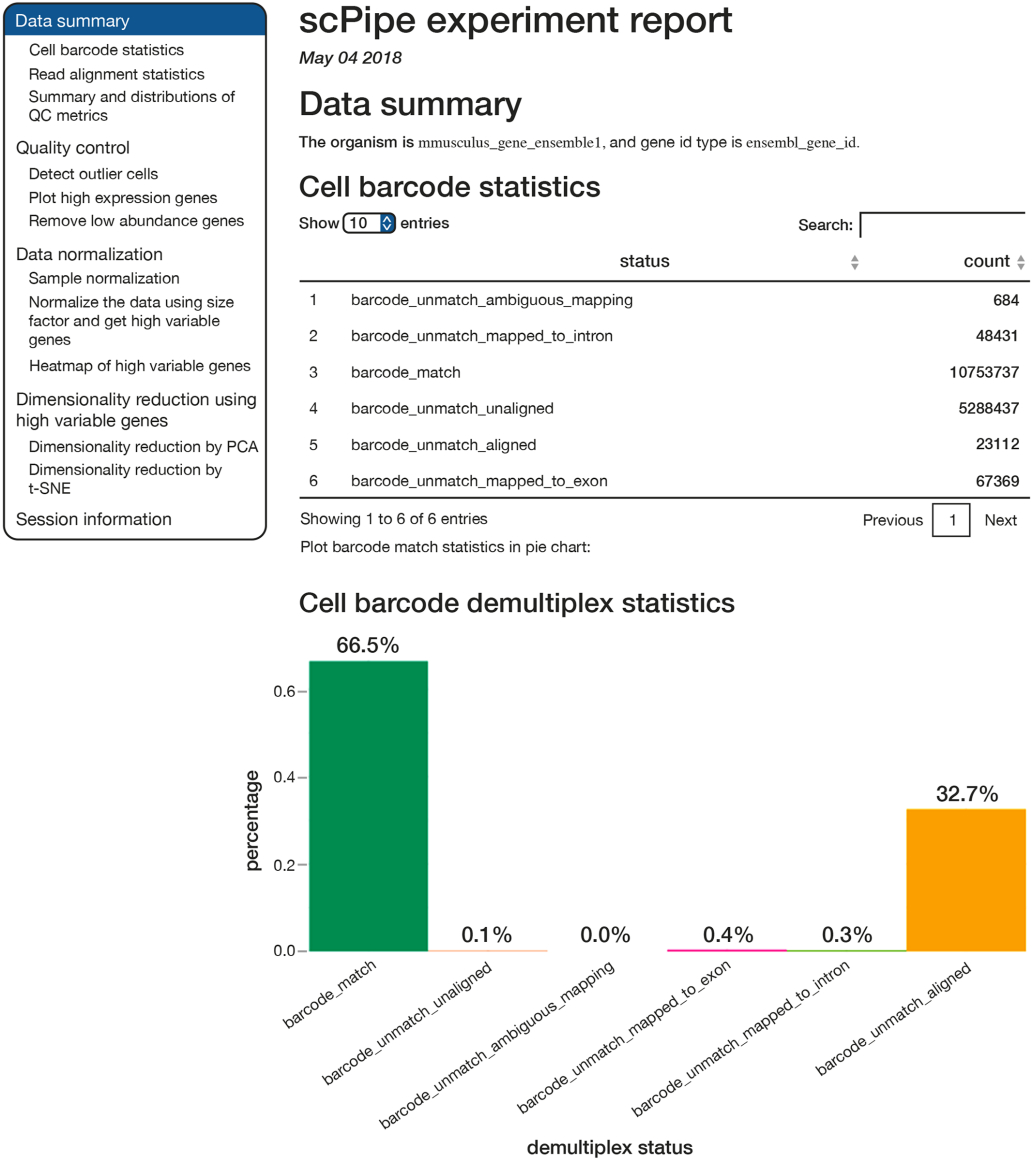
Screenshot of an HTML report created by *scPipe* for the mouse blood cell dataset. This report organises the output of *scPipe*, including run parameters and QC metrics and also generates basic dimension reduction plots of the data. Such reports provide a convenient format for communicating basic QC information to collaborators to help them evaluate the overall quality of an experiment.

An HTML report generated using *rmarkdown* [15] that includes all run parameters used, QC statistics and various types of dimension reduction plots of both the gene expression data and QC metrics can be generated using the create_report function (Fig 4). As *scPipe* generates consistent QC measures across different protocols and experiments, these QC metrics can be easily combined (Fig 2C and 2D) to facilitate comparisons between multiple datasets.

After QC, the data can be easily passed to downstream packages. Use of the *SingleCellExperiment* class ensures the output of *scPipe* is fully interoperable with a range of other Bioconductor packages. For instance, packages such as *scater*, *scran* and *BASiCS* [23] can be used for normalization, *zinbwave* [24] for dealing with zero inflation in the data and removing unwanted variation, *SC3* [25] and *clusterExperiment* [26] for clustering and *MAST* [27] and *scDD* [28] for differential expression analysis.

#### Using *scPipe*

A vignette accompanying the package provides further details on implementation and an example use case on the CEL-seq2 mouse blood dataset.

**Fig 5:**
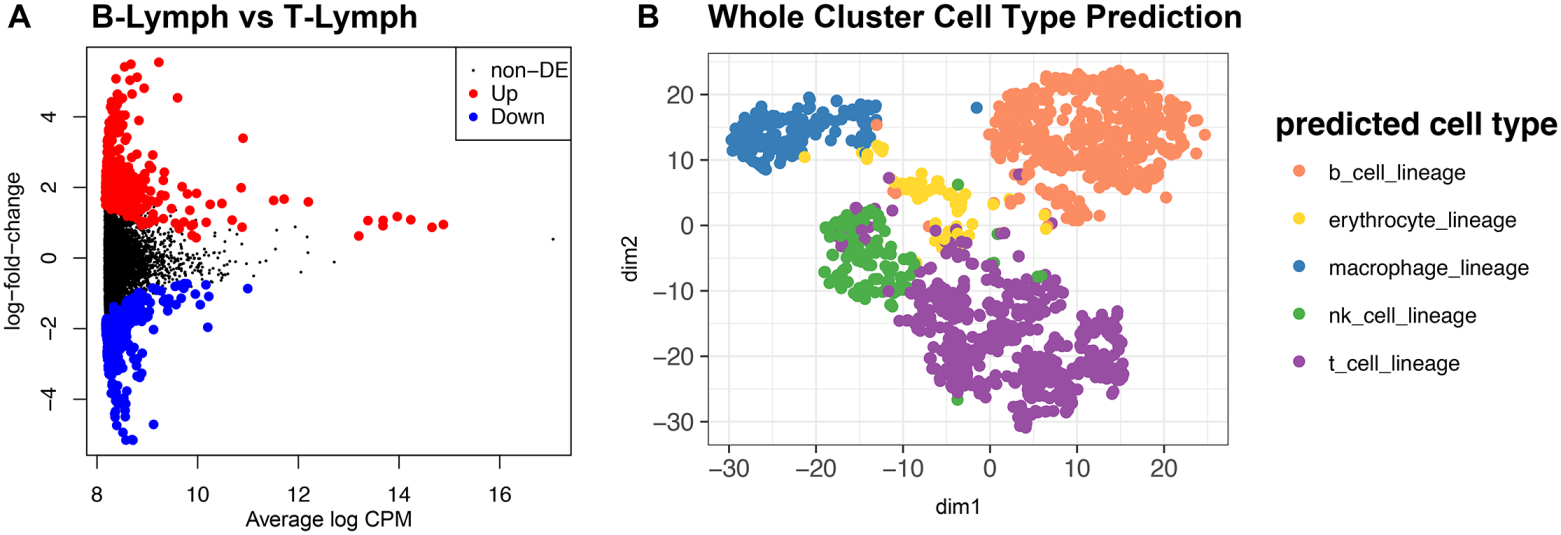
Analysis results produced with *SingleCellExperiment*-compatible packages from Bioconductor. (**A**) Differential gene expression results from comparing B-lymphocytes and T-lymphocytes known *a priori* in the CEL-seq2 mouse blood dataset. Highlighted points are genes determined to be significantly differentially expressed from LRT with 0.05 FDR cutoff. (**B**) tSNE coloured by cell type predictions obtained for *SC3* clusters for the first spleen sample from the Human Cell Atlas ischaemic sensitivity dataset. The 10 clusters from *SC3* were assigned to 5 distinct cell types from the reference set of 12 lineages.

This example dataset contains 100 million reads and takes under 2 hours to process on a standard Linux server. *scPipe* creates a *SingleCellExperiment* object from the count matrix and mapping statistics output by the pipeline which can be manipulated using other Bioconductor packages for single cell analysis. *scPipe*’s outlier detection has been used to remove low quality cells (Fig 3). For the mouse blood dataset, scPipe’s outlier removal retained 359 of 383 cells for further analysis and in the human spleen dataset, 1,397 of the 2,273 detected cells were retained for further analysis. We applied *scran* to compute size factors for normalization. Differential expression analysis was performed between the *a priori* defined B-lymphocyte and T-lymphocyte cells (based on FACS sorting information available for the cells in each well) using a generalised linear model from *edgeR* [29] after performing conversion to a *DGEList* using *scater* (Fig 5A). From this analysis 636 differentially expressed genes were discovered based on likelihood-ratio testing (LRT) at a 0.05 false-discovery-rate (FDR) threshold.

A second example uses data from the pilot Human Cell Atlas ischaemic sensitivity dataset, which was again preprocessed by *scPipe*, with automatic outlier detection leaving 1,397 cells, which were then normalized using *scran*. Next, we performed cell clustering with *SC3*, where the number of clusters produced was optimised by *SC3*’s estimate_k function to give 10 clusters. To annotate these clusters, the mean normalized expression of each cluster was correlated with a microarray dataset of 12 immunological lineages [30]. The lineage with the highest correlation was considered the most likely cell type candidate for each cluster. Using this method, the 10 clusters identified by *SC3* were assigned to 5 of the 12 potential candidate lineages (Fig 5B).

A third example of running *scPipe* on the Chromium 10X cell line data is, which has 400 million reads from around 1,000 cells takes about 10 hours on a standard Linux server. We also provide examples of using *scPipe* with an alternate aligner (STAR [31]). Code for each of these example analyses is provided as Supplementary Information from http://bioinf.wehi.edu.au/scPipe/.

*scPipe* is a fully modular pipeline, with each step generating independent results. It is also flexible with its inputs, accepting FASTQ as outlined above, or BAM files produced by *CellRanger* and Dropseq tools (http://mccarrolllab.com/dropseq/) from which a gene count matrix can be generated. This approach ensures interoperability with tools both inside and outside of Bioconductor to give the data analyst maximum flexibility when configuring their scRNA-seq analysis pipelines.

#### Comparing *scPipe* with other pipelines

We reviewed the tools currently available for scRNA-seq data preprocessing identified through the ‘Alignment’ and ‘UMIs’ categories provided by the https://www.scrna-tools.org/ website [4]. The search revealed 9 tools that have overlapping functionality with *scPipe*, summarised in Table 1. Our software is the only Bioconductor package that spans all major single cell platforms (both plate and droplet-based) and the full range of preprocessing tasks (alignment, UMI handling and QC). The shell program *zUMIs* [32] is another program that covers the breadth of platforms and preprocessing tasks. Other software tools, such as *dropSeqPipe*, *CellRanger* and *dropEst* [8] only deal with data from droplet-based protocols, *Sharq* [33] is suitable for plate-based approaches, and the remaining handle the UMI processing step only. *CellRanger* is the only tool that also offers downstream analysis, with clustering and differential expression testing included in its default output.

**Table 1:** Summary of the data preprocessing software currently available for scRNA-seq analysis and the particular tasks covered by each package.

Software	Language	Input	Multiple technologies	Analysis task			
				Alignment	UMI handling	QC metrics	Downstream analysis
scPipe	R	FASTQ	✓	✓	✓	✓	×
zUMIs	Shell	FASTQ	✓	✓	✓	✓	×
dropSeqPipe	Snakemake	FASTQ	×	✓	✓	✓	×
Sharq	Python/Perl/R	FASTQ	×	✓	✓	✓	×
CellRanger	Python/R	BCL/FASTQ	×	✓	✓	✓	✓
dropEst	C++/R	FASTQ	×	✓	✓	✓	×
UMI-tools	Python	FASTQ	✓	×	✓	×	×
umis	Python	FASTQ	✓	×	✓	×	×
sircel	Python	FASTQ	✓	×	✓	×	×
TRUmiCount	R/Shell	BAM	✓	×	✓	×	×

**Fig 6:**
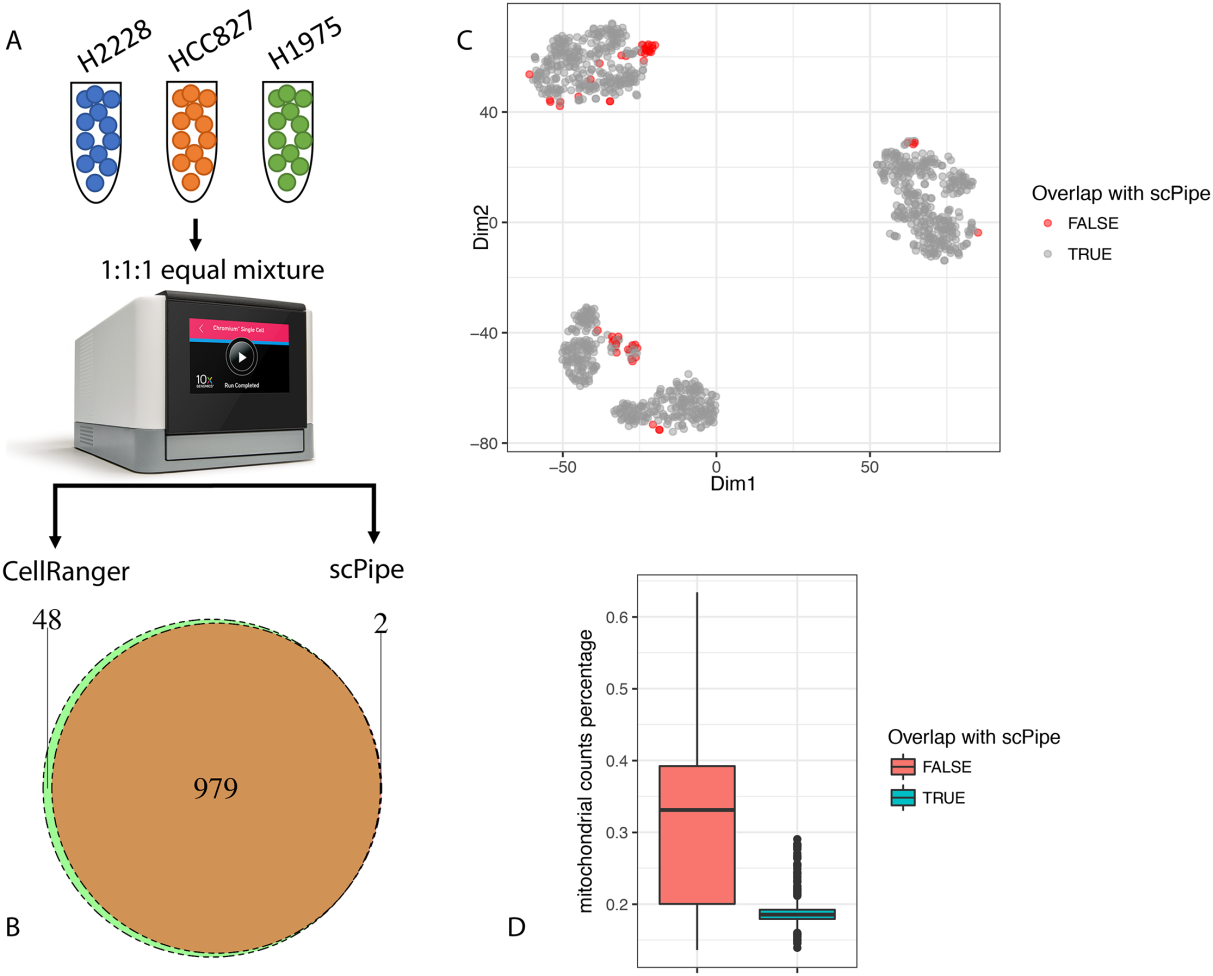
Comparing *scPipe* and *CellRanger*. (**A**) The workflow for the comparison. An equal mixture of cells from three cell lines are sequenced using the Chromium 10X platform (see Materials and methods). Data were processed by *scPipe* and *CellRanger*. (**B**) The pie chart shows the overlap of cell barcode detected by *scPipe* and *CellRanger*. (**C**) The t-SNE plot generated using *CellRanger* output. Cell barcodes that only exist in *CellRanger* are highlighted. (**D**) Box plots showing the percentage of mitochondrial gene counts in cells that overlap with *scPipe* or only exist in the *CellRanger* results.

In order to highlight the differences between *scPipe* and the popular *CellRanger* pipeline that processes raw data from the Chromium 10X platform, we performed a benchmark experiment that included 3 human lung adenocarcinoma cell lines (see Materials and methods) and processed the raw data using both *scPipe* and *CellRanger* (Fig 6A). The dataset contains about 1,000 cells. *CellRanger* returns 1,027 cells and *scPipe* 981 cells after QC (Fig 6B). The t-SNE plot [34] generated from the *CellRanger* output shows that the 48 cells that appear in the *CellRanger* results but not in *scPipe* tend to cluster together (Fig 6C). Inspection of their QC metrics (Fig 6D) shows that these cells have higher proportions of mitochondrial gene counts, suggesting they may be dead cells that should be excluded from downstream analysis. Since *CellRanger* only uses the UMI counts per cell as a QC cutoff, the results generated by *CellRanger* may contain dead cells and benefit from a further round of QC. The *scPipe* analysis on the other hand uses multiple QC metrics by default (Fig 3) to achieve a robust measure of cell quality to ensure low quality cells are discarded. This comparison shows the benefit of *scPipe*’s built-in QC step.

## Availability and future directions

The *scPipe* package is available from https://www.bioconductor.org/packages/scPipe. Code for each of the example analyses described in the ‘*Using scPipe*’ section above is available from http://bioinf.wehi.edu.au/scPipe/. Bug reports and questions about using *scPipe* should be posted on the Bioconductor support site (https://support.bioconductor.org/).

With the growing popularity of scRNA-seq technology, many tools have been developed for normalization, dimensionality reduction and clustering. There are relatively few packages designed to handle the raw data obtained from the various 3’ end sequencing protocols with their associated UMIs and cell-specific barcodes from beginning to end and collect detailed quality control information. The *scPipe* package bridges this gap between the raw FASTQ files with mixed barcode types and transcript sequences and the gene count matrix that is the entry point for all downstream analyses. *scPipe* outputs numerous QC metrics obtained at each preprocessing step and displays these results in an HTML report to assist end users in QC evaluations. Future improvements that are planned for *scPipe* include support for new scRNA-seq protocols as they emerge and parallelization of the various preprocessing steps to enable scalability to larger datasets. We also plan to generate a more comprehensive scRNA-seq benchmark dataset to ensure the default UMI correction and quality control methods used in *scPipe* are optimal that will also allow for a more detailed comparison of *scPipe* with other relevant analysis pipelines, such as *zUMIs*.
